# The role of PLVAP in endothelial cells

**DOI:** 10.1007/s00441-023-03741-1

**Published:** 2023-02-13

**Authors:** Lea Denzer, Walter Muranyi, Horst Schroten, Christian Schwerk

**Affiliations:** Department of Pediatrics, Pediatric Infectious Diseases, Medical Faculty Mannheim, Heidelberg University, 68167 Mannheim, Germany

**Keywords:** Barrier endothelium, Diaphragms, Fenestrae, Non-barrier endothelium, PLVAP

## Abstract

Endothelial cells play a major part in the regulation of vascular permeability and angiogenesis. According to their duty to fit the needs of the underlying tissue, endothelial cells developed different subtypes with specific endothelial microdomains as caveolae, fenestrae and transendothelial channels which regulate nutrient exchange, leukocyte migration, and permeability. These microdomains can exhibit diaphragms that are formed by the endothelial cell-specific protein plasmalemma vesicle-associated protein (PLVAP), the only known protein component of these diaphragms. Several studies displayed an involvement of PLVAP in diseases as cancer, traumatic spinal cord injury, acute ischemic brain disease, transplant glomerulopathy, Norrie disease and diabetic retinopathy. Besides an upregulation of PLVAP expression within these diseases, pro-angiogenic or pro-inflammatory responses were observed. On the other hand, loss of PLVAP in knockout mice leads to premature mortality due to disrupted homeostasis. Generally, PLVAP is considered as a major factor influencing the permeability of endothelial cells and, finally, to be involved in the regulation of vascular permeability. Following these observations, PLVAP is debated as a novel therapeutic target with respect to the different vascular beds and tissues. In this review, we highlight the structure and functions of PLVAP in different endothelial types in health and disease.

## Introduction

The leading cause of death worldwide is represented by non-communicable diseases (NCDs), comprising 73.4% of total death in 2017, which represents an increase of 22.5% since 2007 (Cao et al. 2018, Collaborators 2018). Within this group, the four major NCDs (cardiovascular diseases (CVDs), cancer, chronic respiratory diseases, and diabetes) together caused 12.4 million deaths according to the WHO in 2015, including CVDs with 6.2 million victims at the leading position.

During the course of CVDs endothelial cells (ECs) lining coronary arteries and blood vessels represent an important subject (Carmeliet and Jain 2000). Besides their ability to detect shear stress followed by molecular signaling to change diameter and thickness of the blood vessel wall, ECs also control passaging of immune cells or molecules into and out of the blood, which is dependent on vascular permeability (Yu et al. 2006). To fulfill the different requests across the entire vascular system and even within one vascular bed, a heterogeneous population of cells with different functions builds up the endothelium.

Vascular permeability is on the one hand regulated by the organization of the ECs themselves, e.g. by a loose or tight order combined with the presence of a basement membrane, and on the other hand by the abundance of EC specific microdomains (Aird 2007a; Augustin and Koh 2017; Auvinen et al. 2019; Bosma et al. 2018; Tse and Stan 2010). The diffusion of substrates can be controlled by a thin proteinaceous diaphragm covering these microdomains, which functions as a physical sieve containing the plasmalemma vesicle associated protein (PLVAP), its only known component (Aird 2007a; Augustin and Koh 2017; Auvinen et al. 2019; Bosma et al. 2018; Herrnberger et al. 2014, 2012; Stan et al. 2012; Tse and Stan 2010). It was demonstrated that the development of the cardiovascular system and postnatal physiological processes, as maintaining blood composition and organ homeostasis, strongly rely on PLVAP, which is, therefore, thought to function in the process of vascular permeability (Bosma et al. 2018; Herrnberger et al. 2014, 2012; Stan et al. 2012). In addition, PLVAP has also been described as leukocyte trafficking molecule playing a crucial role in immune surveillance and inflammation, as it is redistributed in cells after a pro-inflammatory stimulus (Keuschnigg et al. 2009).

Because of their strong heterogeneity, ECs can be classified by different strategies. For a recent review presenting classifications into organ-wide ECs (arterial, venous, capillary, and lymphatic ECs) and organ-specific ECs (blood–brain barrier, liver, heart, kidney, and lung ECs), please refer to Prysinda et al. or Hennigs et al. (Hennigs et al. 2021; Przysinda et al. 2020). In addition, there are also enormous differences according to their role in health or disease, which often lead to distinct ECs properties, thus building another category of ECs e.g., tumor specific ECs.

This review will highlight morphological differences of ECs and the different roles of PLVAP expression and regulation to form these different endothelia throughout the human organism, including its role during disease.

## Morphological differences of endothelia

Due to the demands on ECs to react to the needs of the underlying tissue and the surrounding microenvironment, different endothelial types emerged, which exhibit characteristic structural and functional differences that are specific for each organ (Aird 2007a, b; Rhodin 1955; Wisse 1970; Zhou et al. 2014). Consequently, the endothelium may be continuous, fenestrated, or discontinuous (sinusoidal), to differentially control and regulate vascular permeability for water and solutes (Auvinen et al. 2019; Bosma et al. 2018; Tse and Stan 2010). As will be discussed later in this review, PLVAP plays important roles in the manifestation of the different types of endothelia, with its expression and functions strongly varying dependent on the endothelial type.

**Fig. 1:**
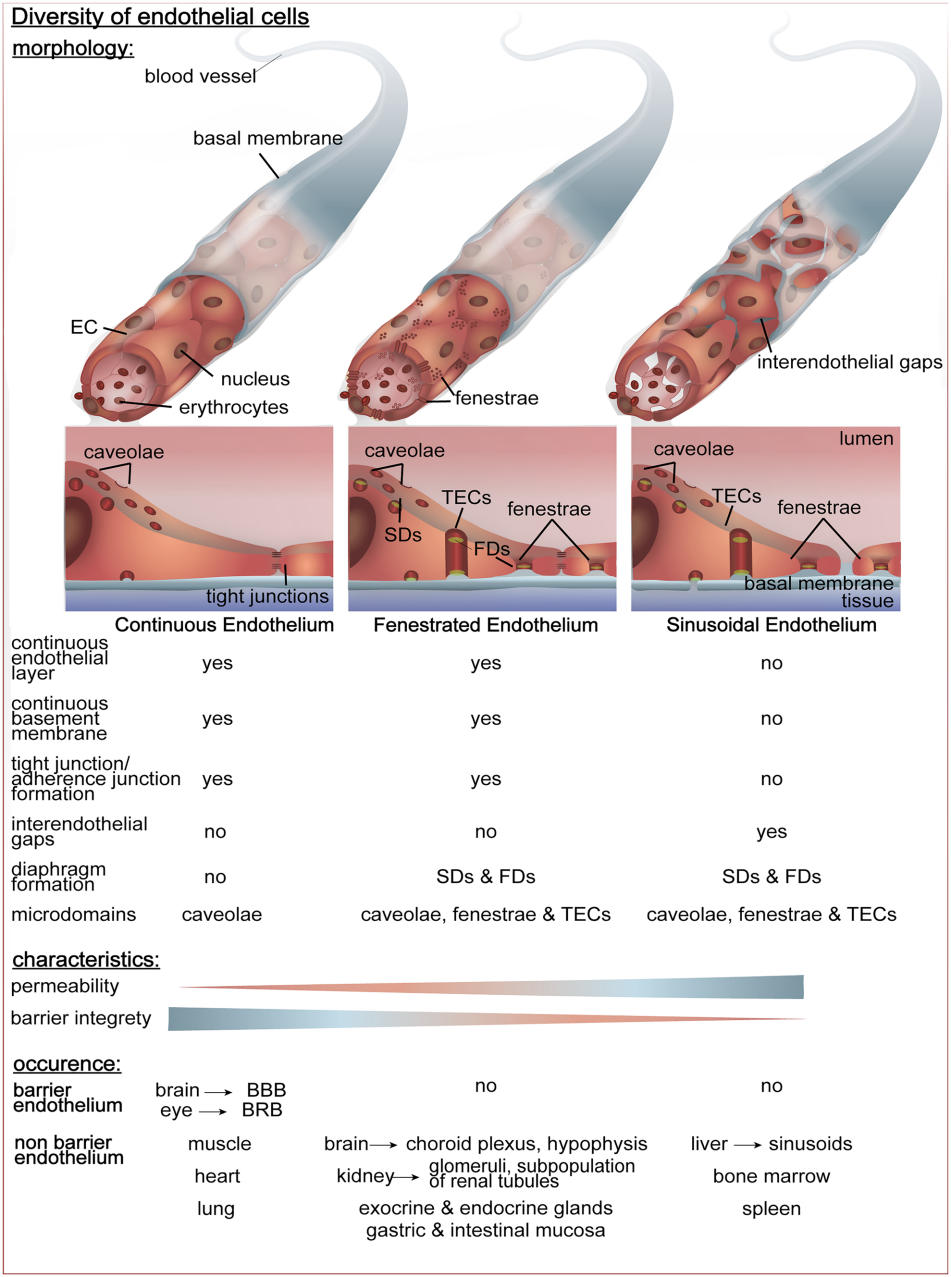
Morphological properties of different endothelial subtypes and their occurrence within the organism. The figure compares the morphology (top), the main characteristics (middle) and the occurrence inside the body (bottom) of the three endothelial types, which are the continuous endothelium, the fenestrated endothelium, and the sinusoidal endothelium

Endothelia that form a diffusion barrier between blood and tissue, thereby ensuring a high selectivity for nutrients, proteins, and immune cells, are organized as the so-called continuous endothelium (see Fig. 1). Only diffusion of water and small molecules to the extravascular compartment is allowed, thereby preventing loss of plasma proteins and blood cells. It is the least permeable type of ECs that is especially pronounced in some organs, e.g. in the brain, where the blood–brain barrier (BBB) is formed (Aird 2007a; Augustin and Koh 2017; Auvinen et al. 2019; Bosma et al. 2018; Tse and Stan 2010). The continuous endothelium is an uninterrupted endothelium with a continuous basal lamina and dense cell-to-cell contacts known as tight junctions, which is found in muscle tissues, heart, lung, brain, and other organs (Okada et al. 2017; Zhou et al. 2014).

Continuous ECs own structures called caveolae, flask-shaped invaginations of the plasma membrane of about 50–100 nm, that display distinct levels of organization in different cell types, clearly detectable by electron microscopy as grape‐like clusters, rosettes, membrane‐bound or detached vesicles, and tubule‐like structures (Filippini et al. 2018; Gordon et al. 2019; Razani et al. 2002). Following these observations, caveolae were hypothesized to be involved in transcytosis (a form of transcellular trafficking of molecules across the endothelial layer), cholesterol transport, potocytosis, and endocytosis (Anderson et al. 1992; Fielding and Fielding 1995; Rothberg et al. 1992; Schnitzer et al. 1996; Simionescu 1983; Smart et al. 1996; Stan 2005). Furthermore, the caveolin family of proteins was identified as molecular marker for caveolae-enriched membranes (Lisanti et al. 1994a, b, 1995; Sargiacomo et al. 1993; Scherer et al. 1997; Tang et al. 1996).

Continuous endothelia that exhibit another subcellular structure, the so-called fenestrae, are classified as fenestrated endothelia (see Fig. 1). Fenestrae are circular membranal openings about 70 nm in diameter cutting through the full thickness of the cell body, which are often spanned across their opening by a thin 5 to 6 nm non-membranous diaphragm, the fenestral diaphragm (FD) (Aird 2007a; Gordon et al. 2019; Okada et al. 2017; Rhodin 1955). Moreover, fenestrae appear to be arranged in clusters with equidistant spacing within one EC, often forming linear or two-dimensional arrays indicated as “sieve plates” (Ioannidou et al. 2006; Rhodin 1962; Simionescu et al. 1976).

The presence of fenestrae allows higher permeability of ECs for small and medium sized molecules, making them important for regulation of endocrine dependent homeostasis (Gordon et al. 2019). Interestingly, caveolae within fenestrated endothelium are also covered by a diaphragm, the stomatal diaphragm (SD). This type of endothelium is characteristic for vascular beds in organs with increased filtration, secretion or increased transendothelial transport as exocrine and endocrine glands, gastric and intestinal mucosa, choroid plexus, glomeruli, and a subpopulation of renal tubules in the kidney (Aird 2007a, 2012; Okada et al. 2017; Takemura et al. 2017; Wisse 1970). Transendothelial channels (TECs) are other microdomains specific for the fenestrated endothelium (see Fig. 1), with two diaphragms that lack heparan sulfate proteoglycan tufts instead of one, as described for fenestrae and are suggested to arise by fusion of caveolae (Hamilton et al. 2019; Herrnberger et al. 2014, 2012; Rippe et al. 2002; Stan et al. 2012). In another hypothesis, TECs are considered as fenestrae precursors, as they intersperse with fenestrae in thinned regions within the ECs. Still, fenestrae and TECs are thought to enable rapid exchange of molecules between the circulation and the underlying tissue, as both represent pore-like structures (Bosma et al. 2018; Tse and Stan 2010).

The sinusoidal endothelium or discontinuous endothelium also exhibits the microdomains mentioned above (see Fig. 1), but it owns larger openings with diameters of about 30–40 μm in the endothelium, resulting from inter-endothelial gaps and an incomplete basement membrane (Aird 2012; Okada et al. 2017; Wisse 1970). This endothelial type is found in organs with the highest degree of vascular permeability as the liver and hematopoietic organs, e.g. the bone marrow and the spleen (Aird 2007a; Augustin and Koh 2017; Auvinen et al. 2019; Bosma et al. 2018; Okada et al. 2017; Tse and Stan 2010).

## Endothelial microdomains in non-fenestrated and fenestrated ECs

Although caveolae are present in non-fenestrated and fenestrated ECs, only the ones present in the latter contain SDs. In contrast, FDs are present in all fenestrated ECs (Aird 2007a; Augustin and Koh 2017; Auvinen et al. 2019; Bosma et al. 2018; Tse and Stan 2010) (Fig. 1). Interestingly, SDs and FDs have differential biochemical properties, even though their composition and structure are supposed to be similar. Moreover, the permeability of the endothelium is not only defined by the molecular size of the molecules, but also by the molecular net charge interacting with the negatively charged surface of ECs (Pelikan et al. 1979; Sawyer and Srinivasan 1972; Simionescu et al. 1981b).

High numbers of anionic sites are characteristic for FDs, thus creating a difference in charge in FDs, whereas they are absent in SDs (Simionescu et al. 1981b). Interestingly, proteases and heparinases can remove the anionic sites on FDs giving rise to the idea of the glycocalyx covering the luminal side of these diaphragms (Simionescu et al. 1981a). Furthermore, primarily heparan sulfate and/or heparin contribute to the anionic sites of FDs, while the acidic sites on the remainder of the ECs are of a more divers chemical nature (Simionescu et al. 1981a).

It is fascinating that the content of anionic sites in FDs appears higher in comparison to the plasma membrane, which is traditionally proven as negatively charged (Sarin 2010). However, the difference in charge causes an impermeability for anionic proteins at FDs. In contrast, SDs are believed to still enable the passage of these anionic proteins, as they miss the selective layer due to charge difference (Simionescu et al. 1981b).

So far, the only established molecular component of SDs and FDs is PLVAP, which is exclusively known to be present in diaphragms (Auvinen et al. 2019; Herrnberger et al. 2012; Stan et al. 1999b, 2012). The highest PLVAP expression is found within organs that are known for the exhibition of fenestrated ECs, as in the lungs, kidneys, spleen, endocrine glands and digestive tract (Deharvengt et al. 2012; Guo et al. 2016). As vascular permeability strongly relies on the presence of diaphragms in fenestrated endothelium, we further want to highlight the role of PLVAP in diaphragm formation and finally permeability regulation.

## The basics of PLVAP

PLVAP is commonly considered to be endothelium‑specific. It represents an antigen for two classic endothelial antibodies, the Mouse endothelial cell antigen (MECA)- 32 (Hallmann et al. 1995) and pathologische anatomie Leiden- endothelium (PAL- E) (Schlingemann et al. 1985). Furthermore, the antibody 174–2, which recognizes a similar antigen distribution as PAL-E, was described during molecular identification of PLVAP (Niemela et al. 2005). MECA‑32 represents the murine variant of PAL‑E, which has been a widely used vascular marker because of the ability of PAL‑E to discriminate between certain subsets of endothelial cells and its specificity for endothelium (Bosma et al. 2018).

PLVAP is essential for the development of FDs and SDs in fenestrated and sinusoidal endothelium (Ioannidou et al. 2006; Stan et al. 2004) but is absent in the non-fenestrated ECs of the BBB, BRB or other ECs with blood-tissue barrier properties, as PAL-E was unable to stain continuous endothelia, as those of the cerebral cortex and the cerebellum, but could stain the fenestrated endothelium of the choroid plexus (Bosma et al. 2018; Schlingemann et al. 1985, 1997, 1998). Furthermore, regulation of basal permeability, leukocyte migration and angiogenesis was reported to involve PLVAP (Carson-Walter et al. 2005; Keuschnigg et al. 2009; Liu et al. 2010; Madden et al. 2004; Minshall and Malik 2006). This is supported by observations under pathological conditions, where PLVAP is expressed also in the BBB and BRB leading to barrier disruption, such as brain ischemia, cancer, and diabetic retinopathy (Carson-Walter et al. 2005; Hofman et al. 2001; Leenstra et al. 1993; Schlingemann et al. 1999; Shue et al. 2008). Considering its association with cancer, traumatic spinal cord injury, transplant glomerulopathy (TG), ischemic brain disease and ocular disease, PLVAP is also investigated as novel therapeutical target, e.g. during cancer therapy (Klaassen et al. 2009; Mozer et al. 2010; Schafer et al. 2009; Wang et al. 2014; Wisniewska-Kruk et al. 2012, 2014; Yamamoto et al. 2007).

### The molecular structure of PLVAP

**Fig. 2:**
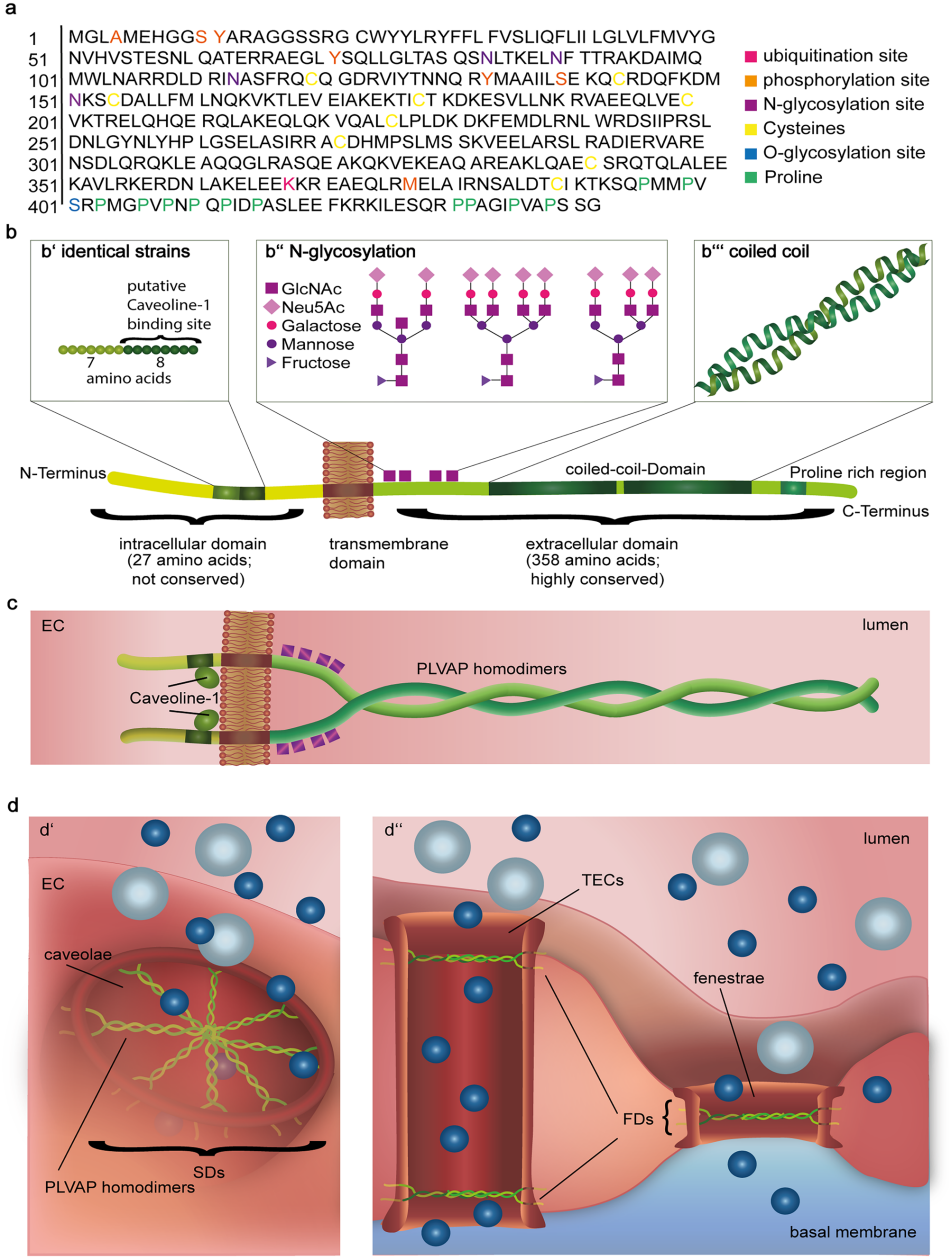
The molecular structure of PLVAP and its arrangement within diaphragms. The figure provides an overview over the molecular structure of PLVAP from **a** its amino acid sequence to **b** its secondary and **c** tertiary structure, showing its integration into the cell membrane. **d** Shows how the homodimers are organized in a wheel-like structure within caveolae, TECs and fenestrae and thus forming SDs (**d’**) or FDs (**d’’**), respectively

PLVAP is a 55–65 kDA type II integral membrane *N*-glycosylated glycoprotein forming homodimers in situ and binds to heparin at physiological pH (Hnasko et al. 2002; Stan 2004; Stan et al. 2001, 1999a, b; Tse and Stan 2010) (see Fig. 2). The molecular structure of PLVAP is made up of a short intracellular domain (27 amino acids), a single span transmembrane domain and a large extracellular domain (380 amino acids) (Stan 2005; Stan et al. 2001, 1999a). There are two short identical stretches of approximately 7–8 amino acids within the non-conserved intracellular domain. One contains a putative caveolin-1 binding domain, counts 8 amino acids, and can be found adjacent to the transmembrane region, while the other counts 7 amino acids and is located at the extreme N-terminus (Couet et al. 1997; Stan 2005). The extracellular domain is highly conserved and consists of four *N*-glycosylation sites, two coiled-coil domains and a proline-rich region near the C-terminus (Stan 2004; Stan et al. 2001). An intermolecular superhelix dominates the whole extracellular domain, giving the protein a rod-like structure (Stan 2007; Stan et al. 2001). Furthermore, PLVAP is postulated to build dimers, which in turn form radial fibrils that are organized in an octagonal wheel-like symmetry to build up FDs or SDs (Bearer and Orci 1985; Rothberg et al. 1992; Stan 2005). Thereby, the extracellular tail of the fibrils interweaves in a central mesh and the intracellular tail anchors the fibrils within the cell membrane. It binds either directly to the cytoskeleton or is fixed to it via cytoskeletal linker molecules (Keuschnigg et al. 2009; Stan 2005). To prevent PLVAP to collapse on the extracellular side, it is glycosylated near the transmembrane domain (Stan et al. 1999b). Considering the wheel-like structure of PLVAP in diaphragms, it is assumed that the distance between adjacent fibrils at the rim is approximately 6 nm (Bearer and Orci 1985; Rantakari et al. 2015).

### The diverse functions of PLVAP

Many endothelia first develop without a functional barrier, which starts to be established during embryogenesis, and then differentiate further to the mature endothelium forming barriers as the BRB and the BBB (Gariano 2003; van der Wijk et al. 2019). These acquired adult phenotypes and properties are reversed under pathological conditions, thus inducing breakdown of the barrier, as it happens during diabetic macular edema (DME) that leads to blindness or in brain tumors (Klaassen et al. 2013; Leenstra et al. 1993). As consequence, there is a high interest in all factors that either build up the functional barrier or lead to its destruction. So far, genes that are involved in the regulation of paracellular transport, such as claudin-5 and occludin, or in the vesicular and transcellular transport, such as caveolin-1 or PLVAP, were identified as most important for BRB or BBB integrity (Klaassen et al. 2009). Although PLVAP is not expressed in the mature functional BBB or BRB, PLVAP was shown to be a crucial regulator of vascular permeability during embryogenesis and postnatal physiological processes, as maintaining blood composition and organ homeostasis (Bosma et al. 2018; Hallmann et al. 1995; Herrnberger et al. 2014, 2012; Risau and Wolburg 1990; Saunders et al. 2012; Schlingemann et al. 1997; Stan et al. 1999a, 2012).

#### PLVAP facilitates the selective passage of molecules

One function of PLVAP is to provide a structural barrier allowing a selected passage of molecules through the endothelial layer. Indeed, in human or mouse cells, when PLVAP expression is knocked down, endothelial diaphragms disappeared (Ioannidou et al. 2006; Stan et al. 2004). Caveolin-1-knockout or cavin-1-knockout mice revealed reduced PLVAP protein levels in tissues, as they lack the necessary structures to form diaphragms. In addition, the reduced PLVAP levels were not due to altered transcription or concentration rates, but due to an increased internalization rate independent from clathrin and dynamin-dependent pathways and lysosomal degradation (Tkachenko et al. 2012). This led to the conclusion that the only structural function of PLVAP is the formation of diaphragms.

A calculation that considers the wheel-like structure of PLVAP, the rim distance between the fibrils, the physiological upper limit pore size of fenestrated endothelium and the underlying lamina basalis revealed increased permeability for plasma proteins of 6 to 30 nm diameter in the absence of PLVAP (Bearer and Orci 1985; Rantakari et al. 2015; Sarin 2010; Stan et al. 2012). Except for large protein complexes or lipoprotein particles, such as chylomicrons, all plasma proteins can, therefore, passage a fenestrated endothelial layer and diffuse into the underlying tissue when PLVAP is not present. Nevertheless, there are hints pointing towards additional factors that are required for diaphragm formation, as successful diaphragm restorage after PLVAP reconstitution in knockdown mice was only observed in ECs with vascular beds that natively form diaphragms (Stan et al. 2012).

#### Structure and stability support

Besides their functions in mature endothelium, diaphragms are considered to structurally stabilize caveolae and fenestrae and to provide mechanical strength to vessels during embryogenesis and postnatal physiological processes (Herrnberger et al. 2012). Aberrant morphological fenestral phenotypes without FDs were detected in PLVAP knockout mice and in an in vitro assay, baring width of 50–120 and 20–400 nm, respectively (Ioannidou et al. 2006; Stan et al. 2012). Transmission electron microscopy of samples obtained from PLVAP knockout mice demonstrated vessels with large openings covered by degranulated thrombocytes resulting from the lack of SDs. Consequently, these mice developed subcutaneous edema, hemorrhages, and cardiac and vascular defects, and died before birth (Herrnberger et al. 2012; Stan et al. 2012).

Additionally, it was shown that PLVAP knockout mice survived postnatally up to 4 weeks or survived up to 3–4 months, respectively, but suffered from growth retardation, anemia, and selective leakage of plasma proteins into the interstitium with subsequent edema and dyslipidemia, eventually leading to a lethal, protein-losing enteropathy (Herrnberger et al. 2012; Rantakari et al. 2015; Stan et al. 2012). Moreover, humans that own a nonsense mutation in the *PLVAP*-gene develop similar disease profiles characterized by protein losing enteropathy, hypoproteinemia, hypoalbuminemia, and hypertriglyceridemia, which can lead to a kwashiorkor‐like wasting syndrome and death (Elkadri et al. 2015; Stan et al. 2012).

However, as there are also caveolae and fenestrae in mature endothelium that lack diaphragms (as present in the kidney glomeruli and liver sinusoids), chances are low that PLVAP gives structural stability as it does in the embryonic endothelium (Stan et al. 2012). Instead, it is possible that controlled permeability is important during the embryogenic development, as *PLVAP* is known to be expressed in these endothelia during embryogenesis and to have a mechanical stabilization function (Ioannidou et al. 2006; Rantakari et al. 2016).

#### Leukocyte trafficking

Within inflammation and immunity, one of the central concepts is the migration of leukocytes from the bloodstream into tissues across the endothelium by a transcellular pathway (Keuschnigg et al. 2009; Liu et al. 2010). During this process, leucocytes were found to be surrounded by rings containing PLVAP and caveolin-1 (Keuschnigg et al. 2009). The presence of caveolin-1 and vimentin (part of the cytoskeleton) seems to redistribute PLVAP leading to a colocalization of all those proteins. In an acute peritonitis model, leukocyte migration was decreased up to 85% when PLVAP was blocked with a MECA-32 antibody, leading to the conclusion that PLVAP enables an increase of leukocyte trafficking possibly intensifying inflammation (Keuschnigg et al. 2009).

Furthermore, PLVAP is also expressed in lymphatic vessels that enable efficient interaction of peripheral antigens with lymphocytes (Germain et al. 2012; Girard et al. 2012; Rantakari et al. 2015). Here, PLVAP is localized in diaphragms of caveolae, TECs and vesiculo-vacuolar organelles (VVOs) within the subcapsular sinus lymphatic ECs (LECs) of lymph nodes, thereby regulating the entry of soluble antigens and lymphocytes into the parenchyma (Rantakari et al. 2015). Thus, an increase of leukocyte transmigration through the sinus floor and non-selective antigen entry into the lymph system could be detected in *PLVAP*-null mice. As consequence, PLVAP was hypothesized to be mandatory for regulation of a selective entry of lymphocytes and antigens into the lymphatic nodes (Rantakari et al. 2015).

#### Diverse functions of PLVAP in barrier and non-barrier endothelium

**Fig. 3:**
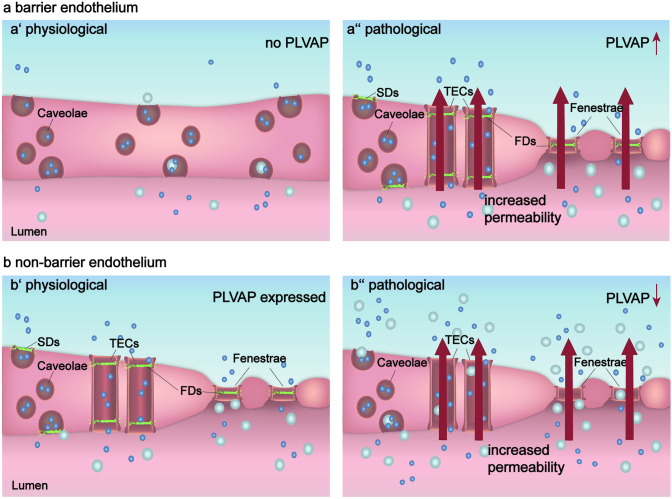
The effect of PLVAP expression on vascular permeability and barrier integrity in barrier endothelium (**a**) and non-barrier endothelium (**b**) under physiological (**a’**, **b’**) and pathological (**a’’**, **b’’**) conditions

The functions of PLVAP differ according to their expression in fenestrated or non-fenestrated endothelia. PLVAP is thought to prevent excessive protein leakage from the bloodstream into the tissue in non-barrier endothelium and is, therefore, considered as regulator for the fine tuning of vascular permeability (van der Wijk et al. 2019). In contrast, PLVAP has a reverse role in barrier endothelium, as in these endothelia it is responsible for loss of barrier integrity, protein leakage and barrier breakdown (Wisniewska-Kruk et al. 2016), and is considered to be expressed in mature barrier endothelium only under pathological conditions (van der Wijk et al. 2019). The expression and functions of PLVAP in barrier and non-barrier endothelia are summarized in Fig. 3.

##### Functions in barrier endothelium

In barrier-forming endothelium, *PLVAP* expression is only found during the embryogenesis or in postnatal development, but not in mature endothelium (Daneman et al. 2010; Hallmann et al. 1995; Liebner and Plate 2010; Schlingemann et al. 1997; Umans et al. 2017; van der Wijk et al. 2019). In the BBB and BRB, *PLVAP* expression decreases with the progression of vasculature maturation and development of a functional barrier (Daneman et al. 2010; Hallmann et al. 1995; Liebner and Plate 2010; Umans et al. 2017). Thus, its expression is only reinduced under pathological conditions leading to vascular leakage, as found during diabetic retinopathy (Hofman et al. 2001; Schlingemann et al. 1999; Witmer et al. 2002), brain ischemia (Carson-Walter et al. 2005; Shue et al. 2008) and brain tumors (Schlingemann et al. 1985; Shue et al. 2008). A trans-endothelial electrical resistance assay revealed reduced loss of BRB integrity after vascular endothelial growth factor (VEGF) treatment, when *PLVAP* expression was inhibited using short hairpin RNAs (shRNA) (Wisniewska-Kruk et al. 2016). Another observation was the reduced permeability for 70 kDa tracers, but not for 766 Da tracers, in an in vitro model of retinal leakage and in a hypoxia-induced retinopathy (OIR) mouse model after VEGF induction due to PLVAP inhibition (Wisniewska-Kruk et al. 2012, 2016).

These results imply a crucial role for PLVAP in VEGF-induced leakage via the transcellular pathway, allowing increased passage of large molecules, while having a low effect on small molecules that are known to use the paracellular transport. Furthermore, VEGF-induced caveolae formation was reduced back to basal levels by PLVAP knockdown in human retinal explants, leading to the conclusion that PLVAP enables the formation of caveolae, which probably possess SDs containing PLVAP and thereby induce leakage (Wisniewska-Kruk et al. 2016). In *PLVAP*-deficient mice, lack of *PLVAP* expression had no influence on the basal caveolae number, but resulted in a complete lack of all diaphragms indicating that PLVAP enables the formation of caveolae, which are probably covered by SDs containing PLVAP, and thus facilitate permeability and leakage (Bosma et al. 2018; Herrnberger et al. 2012; Wisniewska-Kruk et al. 2016). One theory implies PLVAP to increase basal VEGFR2 availability in caveolae in a primary or secondary mechanism and, thereby, modulating vascular permeability and caveolae formation (Labrecque et al. 2003; Tahir et al. 2009). As PLVAP interacts with NRP-1, an important co-receptor during VEGFR2 signaling that regulates the surface expression of VEGFR2, PLVAP could stabilize the VEGFR2/NRP-1 complex, thereby facilitating VEGFR2 signaling, which in turn induces *PLVAP* expression completing a positive feedback loop (Gelfand et al. 2014; Keuschnigg et al. 2009; Strickland et al. 2005). As interesting the possibility of a positive feedback loop resulting in breakdown of the BBB or the BRB is, this theory has still to be elucidated.

There may also be a possible functional role of PLVAP in the paracellular pathway in barrier endothelium (already discussed above), as it is involved in the regulation of the fenestrae and caveolae number within a cell (Herrnberger et al. 2014, 2012; Wisniewska-Kruk et al. 2016). This event is believed to include reorganization of the cytoskeletal framework, but more research in this area is needed, as the precise molecular mechanisms leading to the biogenesis of caveolae and fenestrae are still unknown (Ioannidou et al. 2006). However, PLVAP may regulate intercellular gap formation, as VEGF stimulation significantly reduced stress fiber formation in bovine retinal endothelial cells, when PLVAP was inhibited (Wisniewska-Kruk et al. 2016). In unstimulated cells, stress fiber formation was only affected in a limited degree after PLVAP inhibition. Therefore, VEGF is believed to play a central role in this process, while PLVAP is indirectly participating, maybe via its suspected link to actin-binding proteins (Wisniewska-Kruk et al. 2016). Moreover, VEGF-A was shown to induce Rho-A, which owns functions during stress fiber formation and cellular contractility (Bryan et al. 2010; Chrzanowska-Wodnicka and Burridge 1996). Since *PLVAP* knockdown significantly blocked VEGF-induced caveolae formation, PLVAP may induce VEGFR2 dependent Rho GTPase signaling in cells to modulate stress fiber formation (Bosma et al. 2018; Wisniewska-Kruk et al. 2016). The hypothesis of PLVAP affecting paracellular transport is further supported by altered expression of tight junctions and adherens junctions in heterozygous *PLVAP* mice, even if it is still not known how these altered expression levels manifest into functional junction characteristics (Bosma et al. 2018; van der Wijk et al. 2019). As consequence, more research is needed on the field of transcellular and paracellular transport in barrier endothelium and the mechanisms how PLVAP modulates both in pathological conditions. Potentially, this research will finally give rise for new therapeutical targets to fight pathological conditions favoring breakage of barriers as the BBB or BRB.

##### PLVAP in non-barrier endothelium reduces vascular permeability

In contrast to barrier endothelium, *PLVAP* is expressed in non-barrier endothelium also in the mature endothelium. This type of endothelium includes fenestrated endothelia covered by FDs (except the kidney glomerulus and liver sinusoidal capillaries, which lack FDs), and the continuous endothelia of skin, muscle, and lung (Niemela et al. 2005; Schlingemann et al. 1998; Stan et al. 1999b, 2012).

Studies with *PLVAP* knockout mice revealed significantly reduced albumin and albumin/globulin ratios with minimal electrolyte imbalance compared to littermates (Stan et al. 1999b). A following Evans Blue dye extravasation assay exposed significant leakage of plasma proteins in organs with fenestrated capillaries as intestine, kidney, and pancreas, suggesting that the reduced albumin and albumin/globulin ratios were not due to decreased protein production, enhanced catabolism, or nephropathy. In organs with continuous endothelium as heart muscle and lung, only a minimal increase in the vascular leakage was detected, while there was no effect in liver sinusoids and glomeruli (Stan et al. 1999b). Therefore, barrier integrity in the ECs of the kidney glomerulus lacking FDs is not altered due to PLVAP deletion. Thus, the observed hypoproteinemia and hypoalbuminemia in *PLVAP*-knockout mice are the consequence of increased vascular leakage in fenestrated endothelium (Stan et al. 1999b). Two case studies with infants owning a homozygous non-sense mutation in the *PLVAP* gene supported these results, as the infants died at 5 months and 15 days of age, because *PLVAP* was targeted for degradation by non-sense mediated mRNA decay, leading to complete absence of SDs and FDs in endothelial cells (Broekaert et al. 2018; Elkadri et al. 2015). As result, the patients suffered from similar phenotypes as observed in the *PLVAP*-knockout mice; severe protein-losing enteropathy, characterized by excessive loss of plasma proteins in the gastrointestinal tract, a severe pathological condition that can eventually lead to death (Broekaert et al. 2018; Elkadri et al. 2015). All observations taken together support the hypothesis that loss of *PLVAP* expression in fenestrated endothelium results in vascular leakage of proteins within the corresponding vessels. Considering PLVAP localization in diaphragms, it was concluded that it owns filter functions for fenestrae or caveolae to regulate vascular permeability, as it provides size limitation for macromolecules, but allows the passage of water and solutes.

VEC*-PV1HA* transgenic mice expressing *PLVAP* tagged with human influenza hemagglutinin (HA) (PV1HA) under the control of the vascular endothelial-cadherin promoter and a 5′-intronic enhancer element demonstrated ~ 30–50% reconstitution of native *PLVAP* expression levels in their tissues (Hisatsune et al. 2005; Stan et al. 2012). Furthermore, PV1^−/−^(*PV1*^*ECRC*^) knockout mice that expressed the *VEC-PV1HA*^+*/tg*^ construct also displayed ~ 30–50% reconstitution of PV1HA, which significantly increased the survival of *PV1*^*ECRC*^ mice up to 60% of expected Mendelian frequency, on a mixed background. Additionally, FDs or SDs in the lungs, adrenal glands, kidneys, pancreas, thyroid and intestine were reestablished, which led to improved survival, further indicating a crucial role for PLVAP in the regulation of vascular permeability (Guo et al. 2016; Stan et al. 2012).

#### In different vessels, permeability of SDs and FDs are determined by biochemical and morphological properties

Considering the differential biochemical properties of the distinct types of diaphragms, it is hypothesized that PLVAP binds to several glycoproteins in various vascular beds, which could alter the chemical composition of the endothelial opening and as consequence the selectivity towards molecules (Bosma et al. 2018). Contrarily, the possibility exists that the difference between SDs and FDs is a result of additional proteins, which might be present in the diaphragms besides PLVAP. However, Stan et al. reported no increased permeability in continuous endothelium of the lung, which normally exhibits only caveolae with SDs, in *PLVAP*-knockout mice (Stan et al. 2012). Therefore, the authors concluded that the leakage of plasma proteins via caveolae was not directly induced by the absence of PLVAP. Indeed, heterozygous *PLVAP* mice showed similar basal vascular permeability for proteins in the continuous endothelium of the dorsal skin as their WT littermates (van der Wijk et al. 2019).

Thus, the impact of PLVAP loss in SDs and FDs in caveolae and fenestrae, respectively, might be due to their morphological properties. Without diaphragms as permselective barrier, fenestrae would form pores connecting the capillary lumen with the underlying tissue enabling uncontrolled diffusion of macromolecules. Lacking diaphragms at caveolae will facilitate the entry of macromolecules into vesicles, but it does not affect vesicular trafficking as this requires other proteins as caveolin-1, dynamins and SNARE proteins regulating the formation, scission, and fusion of caveolae, respectively (Chen and Scheller 2001; Fra et al. 1995; Praefcke and McMahon 2004). Another explanation for the prominent leakage in fenestrated endothelium lacking FDs is the natural repellence against anionic proteins that is lost, when FDs are missing (Bosma et al. 2018).

In contrast, *PLVAP* knockdown resulted in a twofold increase in transendothelial electrical resistance and decreased permeability for both 70 kDa tracers and 766 Da tracers in HUVECs, which normally form a relatively permeable barrier without expression of fenestrae and TECS in situ (Stan 2004; Wisniewska-Kruk et al. 2016). Therefore, barrier-like properties seemed to be established in non-barrier endothelium due to missing *PLVAP* expression, including an exceptionally low rate of vesicular transcytosis. This is proposed to counteract the increased entry of macromolecules into caveolae lacking SDs in continuous endothelia (Bosma et al. 2018).

#### PLVAP expression in brain endothelium besides the BBB and BRB

Besides the already documented role of PLVAP in the BBB and BRB, new studies in zebrafish showed the dependency of accurate permeability acquisition in the fenestrated capillaries of the hypophysis on *PLVAP* ortholog expression (Gordon et al. 2019). The neurohypophysis together with the median eminence form the hypothalamo-hypophyseal system, also known as circumventricular organs (CVO) that are located around the midlines of the brain ventricles (Anbalagan et al. 2018; Ganong 2000; Gutnick et al. 2011; Miyata 2015). To regulate homeostasis by recognition of blood-borne proteins and release of neurohormones, the capillaries of the CVOs are fenestrated allowing this major neuroendocrine interface a bidirectional communication between the CNS and the periphery without disrupting the BBB (Anbalagan et al. 2018; Ciofi et al. 2009; Ganong 2000; Gutnick et al. 2011; Miyata 2015; Schaeffer et al. 2014). PLVAP was proven to regulate the rate of blood-borne protein transfer through fenestrated endothelia into the hypophysis, while establishing a permeability boundary between the hypophysis and BBB-containing vasculature (Gordon et al. 2019). However, the regulatory mechanisms of *PLVAP* expression in the ECs of the choroid plexus, another non-barrier endothelium that is known to possess fenestrated ECs, are still not clear (Aird 2007a, 2012; Okada et al. 2017; Wisse 1970).

Since the morphology and function of the choroid plexus endothelium, with its characteristically expressed gene pattern, is different to other ECs, the ECs of the choroid plexus are clearly distinguishable from most other EC populations of the body. In a study with normal human and animal eyes, only weak PAL-E staining was observed in the retina and nerve fiber layer, mostly in the peripapillary area. Still, pronounced staining of capillaries and venules with PAL-E was observed in other structures of the eye (e.g. conjunctiva, episclera, sclera) and the optic nerve head (Schlingemann et al. 1997).

A promising insight into the function of PLVAP may be provided by studies using a novel cell line gained by immortalization of human choroid plexus endothelial cells (iHCPEnC) via ectopic expression of the catalytic unit of the human telomerase (hTERT) (Muranyi et al. 2022). This cell line exhibits fenestrations and FDs and could provide important information about the biologics of *PLVAP* expression in the human choroid plexus.

### Regulation of PLVAP expression

Despite its importance, not much is known about PLVAP and diaphragm regulation (Hamilton et al. 2019). Recently, *PLVAP* expression and fenestration of primary liver sinusoidal endothelial cells (LSECs) were shown to be regulated by Bone morphogenetic protein 9 (BMP9). It was proven that this circulating factor produced by hepatic stellate cells is a key paracrine regulator of liver homeostasis, as it protects against perivascular hepatic fibrosis and controls LSEC (Desroches-Castan et al. 2019). Finally, PLVAP is considered to function downstream of multiple molecules that can induce permeability (Bosma et al. 2018; Daneman et al. 2010; Hallmann et al. 1995; Liebner and Plate 2010; Umans et al. 2017; van der Wijk et al. 2019). Besides angiotensin-2 (Bodor et al. 2012), PMA (Stan et al. 2004), Norrin/Wnt mediated β-catenin signaling (Chen et al. 2012; Liebner et al. 2008; Liebner and Plate 2010; Schafer et al. 2009), Notch-signaling, transforming growth factor-β (TGF-β) (Farber et al. 2018; Mintet et al. 2017), and inflammatory mediators, such as tumor necrosis factor-α (TNF-α) and shear stress (Wasserman et al. 2002), VEGF is reported to be the main regulator of PLVAP (Bodor et al. 2012; Hofman et al. 2001; Klaassen et al. 2009; Strickland et al. 2005; Wisniewska-Kruk et al. 2014). For example, reduced PLVAP levels were able to protect continuous endothelium of the dorsal skin from vascular permeability that is induced by VEGF and histamine stimulation (van der Wijk et al. 2019). Here, we want to highlight the regulatory mechanisms facilitated by VEGF via mitogen activated protein kinase (MAPK) signaling and Norrin/Wnt mediated β-catenin signaling.

#### PLVAP expression regulation via VEGF

Originally, VEGF was described as vascular permeability factor, but it is nowadays also known as potent inducer of angiogenesis (Ferrara and Henzel 1989; Senger et al. 1983). Furthermore, injections of VEGF-induced *PLVAP* expression in retinal vessels in monkey eyes (Hofman et al. 2001). This corresponds with other studies describing increased PLVAP mRNA and protein levels in photoreceptors of transiently overexpressing VEGF mice or VEGF-stimulated bovine retinal endothelial cells (Klaassen et al. 2009; Wisniewska-Kruk et al. 2014). Within the group of VEGFs, VEGF-A was reported to induce new vessels exhibiting fenestrae with diaphragms in a RAC1-dependent manner and was thus concluded to be essential for formation and maintenance of fenestrae and diaphragms (Cao et al. 2001, 2004; Eriksson et al. 2003; Nagy et al. 2006; Roberts et al. 1998; Roberts and Palade 1995).

There are three different tyrosine kinase receptors termed VEGF receptor 1–3 (VEGFR1–3) that bind VEGF and transmit the incoming signals. Systemically delivered VEGFR2 inhibitors or deletion of VEGF-A in the kidney podocytes, pancreas epithelial cells or hepatocytes, caused loss of fenestrae in mice (Carpenter et al. 2005; Eremina et al. 2003; Kamba et al. 2006; Lammert et al. 2003). In addition, selective receptor-specific engineered variants of VEGF-exposed VEGFR2 to be responsible for PLVAP upregulation (Strickland et al. 2005). Thereby, the modulation of *PLVAP* expression as consequence of VEGF‐A/VEGFR2 signaling seems to be context-dependent, as *PLVAP* expression in HUVEC was up-regulated by VEGF-A/VEGFR2 signaling, while it was unchanged or decreased in immortalized mouse EC lines constitutively expressing *PLVAP* (Hnasko et al. 2006a; Ioannidou et al. 2006; Strickland et al. 2005). Moreover, culturing of primary ECs in the presence of VEGF-A resulted in no or poor expression of PLVAP, and inhibition of VEGFR2 signaling in vivo revealed no modification of *PLVAP* expression in lung tissue (Hnasko et al. 2006b; Stan 2004). It should be mentioned that instead of PAL-E or MECA-32, the authors used a goat-antimouse peptide serum against PLVAP. The use of inhibitors for phosphatidylinositol 3-kinase (PI3K) (LY294002) or p38 mitogen-activated protein kinase (p38MAPK) (SB203580) revealed the involvement of P13K- or p38MAPK signaling in VEGFR2-induced *PLVAP* expression, respectively (Strickland et al. 2005). Interestingly, VEGF-A signaling through VEGFR2 is necessary, but not sufficient, to induce PLVAP upregulation, as pharmacological VEGFR2 inhibition or inhibition using antibodies and siRNA did only partly influence its expression (Hamilton et al. 2019). Thus, VEGF-A acts synergistically with other secreted proteins in an MEK1/Erk1/2-dependent manner to upregulate PLVAP.

There are hints that other proteins than VEGF would be able to affect VEGFR2-mediated *PLVAP* expression. Caveolin-1 may be such a protein, as PLVAP protein levels were increased in the lungs of caveolin-1-knockout mice after treatment with an VEGFR2 inhibitor. This effect was not visible in caveolin-2-null mice or WT mice indicating a VEGF-mediated negatively regulated PLVAP expression in the lungs of caveolin-1-null mice (Hnasko et al. 2006b).

However, the way increased VEGF expression affects PLVAP expression may also be dependent on the different organs and species (Hnasko et al. 2006b; Kim et al. 2020; Strickland et al. 2005). Thus, specific combinations of VEGFs are required for selective fenestrated vessel formation in the zebrafish myelencephalic choroid plexus. Accordingly, the combined loss of *VEGFAB*, *VEGFC*, and *VEGFD* causes severely impaired vascularization, while having no effect on the formation of non-fenestrated neighboring vessels. Therefore, the authors concluded specific angiogenic requirements for vascular fenestrated ECs (Parab et al. 2021).

#### Phorbol esters inducing PLVAP expression

Addition of Phorbol esters such as phorbol myristate acetate (PMA), an activator of protein kinase C and a known secretagogue in human ECs, to primary ECs in culture can induce strong de novo formation of fenestrae and TECS, including appropriate diaphragm development (Loesberg et al. 1983; Lombardi et al. 1987; Stan 2004). Furthermore, induction of SDs and PLVAP expression after PMA stimulation was observed in a MEK1/Erk1/2 MAPK‐dependent, and JNK‐, p38‐, PI3K‐, Akt- and PKC‐independent manner (Hamilton et al. 2019; Stan et al. 2004). Since the observed PMA-induced upregulation of PLVAP happened in a dose-dependent and time-dependent manner, the Erk1/2 signaling pathway was postulated to activate PLVAP expression (Stan 2004).

#### The canonical Wnt signaling pathway as regulator of PLVAP expression

Especially in the brain, another important regulator of *PLVAP* expression is the canonical Wnt signaling pathway implicated in angiogenesis and differentiation (Cattelino et al. 2003; Daneman et al. 2009; Liebner et al. 2008; Stenman et al. 2008). It is activated by ligand binding to a Frizzled receptor at its cysteine-rich domain (CRD). This leads to complex formation with LDL-receptor related protein 5 or 6 (LRP5 or LRP6), and cytoplasmic β-catenin degradation is inhibited (Engelhardt and Liebner 2014; Liebner et al. 2008; Moon 2005; Paes et al. 2011; Schafer et al. 2009). Following nuclear translocation of activated, dephosphorylated β-catenin, transcription of genes involved in the regulation of cellular activities, as proliferation, migration, and differentiation, is activated by promotion of the TCF/LEF-1 transcription factor complex (Paes et al. 2011; van Amerongen and Nusse 2009). In addition, the secreted protein Norrin (NDP) was observed to activate Wnt/β-catenin signaling despite being no prototypical Wnt family member (Junge et al. 2009; Ye et al. 2009). Accordingly, it binds a receptor complex uniting Frizzled-4 (FZD4), LRP5, and Tetraspanin-12 (TSPAN12) during development, finally influencing retinal angiogenesis in both mice and humans (Ye et al. 2010). Besides the observation of PLVAP upregulation in developing retinal vascular networks after inhibition of FZD4, treatment with the antibody 1.99.25 antagonizing Norrin- and WNT3A-induced β-catenin accumulation, induced *PLVAP* expression in the deep capillary bed within the adult neural retina, increasing the permeability of the BRB (Paes et al. 2011).

Interestingly, different sensitivities of the vasculature to perturbations in canonical Wnt signaling were observed, depending on the various CNS regions investigated, and revealed the requirement of Wnt signaling to maintain plasticity of barrier properties in the mature CNS vasculature (Zhou et al. 2014). Thus, stabilization of β-catenin enhanced impaired Norrin/Frizzled4 signaling and the subsequent vascular defects in brain and retina. Furthermore, vascular development and barrier defects occurring after a loss of receptor, coreceptor, or ligand were recapped by inhibition of β-catenin–dependent transcription (Zhou et al. 2014). Taken together, these data support the role of the Wnt/β-catenin pathway as crucial regulator during brain angiogenesis and postnatal vascular maturation, and finally establishment of the BBB (Engelhardt and Liebner 2014; Liebner et al. 2008; Paes et al. 2011; Schafer et al. 2009). Moreover, Wnt/β-catenin signaling is also involved in maintenance of barrier properties in the adult brain (Moro et al. 2012; Wang et al. 2012). Also, Wnt and Notch signaling pathways appear to control the downregulation of PLVAP in specialized vascular beds belonging to the BBB, developing arteries and glomeruli, or even in cell culture (Farber et al. 2018; Hnasko et al. 2006b; Liebner et al. 2008; Mintet et al. 2017; Paes et al. 2011; Schafer et al. 2009; Zhou et al. 2014). However, considering the impact of the different vascular beds and microenvironments, much research is required to completely understand the regulation of *PLVAP* expression and its influence on endothelial fenestrations, which in the future might contribute to discover therapeutic strategies to compete the different facets of cardiovascular diseases.

## Role of endothelial cells and PLVAP expression in health and disease

Unfortunately, the heterogeneity of ECs triggers unspecific and unintended targets, especially in disease treatment, e.g. the intended effect of re-fenestration to counter liver disease causes also unintended fenestration and thus permeability in the BBB leading to severe edema (Aird 2007a, b). Hence, a protective effect in one vascular bed could simultaneously cause a deleterious effect in another. Therefore, it is challenging to find a drug or treatment with the required specificity to only affect the intended local vascular bed. In this context, the role of PLVAP should also be considered according to the vascular bed it is expressed in. PLVAP is considered important for many diseases, as it controls the development of SDs and FDs, finally regulating a size-dependent exchange of soluble molecules between the blood plasma and interstitial fluid (see Table 1) (Germain et al. 2012; Girard et al. 2012; Rantakari et al. 2015). In this context, PLVAP upregulation was detected in several pathophysiological processes including angiogenesis, tumorigenesis or the secondary injury of neurons following spinal cord injury (Madden et al. 2004; Mozer et al. 2010). These observations brought PLVAP into the focus as a new therapeutical target. In cancer therapy, the administration of PLVAP antibodies had minimal systemic toxicity, while effectively suppressing tumor growth by induction of vascular thrombosis and extensive necrosis of hepatocellular carcinoma (Wang et al. 2014). In addition, the usage of small hairpin RNA for *PLVAP* downregulation prevented the development of pancreatic adenocarcinoma in xenografts (Deharvengt et al. 2012).

**Table 1:** Role of PLVAP in diseases

	*Cancer*	*Traumatic spinal cord injury*	Transplant glomerulopathy (*TG)*	*Acute ischemic brain disease*	*Norrie disease*	*Diabetic retinopathy (DR)*
*Impact on ECs*	Increased fenestration, malperfusion and hyperpermeability	Rapid loose of ECs and neurons Inflammation due to extensive cell death (Benton et al. 2008; Casella et al. 2006)	Increased numbers of caveolae in glomerular ECs (Yamamoto et al. 2007)	Increased permeability of the microvasculature Fluid accumulation in the extracellular space Brain ischemia, hypoxia and eventually mortality (Shue et al. 2008)	Abnormal angiogenesis Exudative vitreoretinopathy Reduced retinal capillarization (Black et al. 1999; Chen et al. 1993; Luhmann et al. 2005; Shastry et al. 1997)	Increased retinal VEGF levels Loss of blood-retinal barrier (BRB) integrity (Klaassen et al. 2009; Wisniewska-Kruk et al. 2012, 2014)
Occurrence	Brain, lungs, breasts, stomach, liver, pancreas, colon, small intestine, kidneys, ovaries, prostate, uterus, skin and lymph nodes (Madden et al. 2004; Strickland et al. 2005; Tichauer et al. 2014)	Spinal cord (Benton et al. 2008; Casella et al. 2006)	Mature glomerular endothelium (Ichimura et al. 2008; Yamamoto et al. 2007)	Blood–brain barrier (Shue et al. 2008)	Retina, ear (Black et al. 1999; Chen et al. 1993; Shastry et al. 1997)	Diabetic rodents, primates, and humans (Hofman et al. 2001; Klaassen et al. 2009; Wisniewska-Kruk et al. 2012)
Differential expression of PLVAP	Upregulated in primary and metastatic tumors (Liu et al. 2010)	Upregulated (Mozer et al. 2010)	Upregulated (Yamamoto et al. 2007)	PLVAP upregulation (Carson-Walter et al. 2005)	Ectopic expression (Schafer et al. 2009)	Increased levels in caveolae following VEGF exposure (Hofman et al. 2000; Klaassen et al. 2009; Wisniewska-Kruk et al. 2012)
Induction of PLVAP expression	In large, well-vascularized tumors (Carson-Walter et al. 2005)	In neovasculature 12 h post-SCI (Mozer et al. 2010)	Compensatory mechanism (Yamamoto et al. 2007)	Earliest 48 h following disease onset (Shue et al. 2008)	Mutations in the Norrie disease pseudoglioma (*NDP*) gene (Black et al. 1999; Chen et al. 1993; Shastry et al. 1997)	At 72 h following VEGF treatment in bovine retinal ECs (BRECs) (Wisniewska-Kruk et al. 2012)
Impact of PLVAP expression	Facilitates vascular growth in cancer Angiogenesis (Carson-Walter et al. 2005; Madden et al. 2004)	Disruption of neurovascular integrity Inflammation and deterioration of neurovascular function (Mozer et al. 2010)	Permeability increase of ECs to macromolecules Post-injury vascular remodeling (Ichimura et al. 2008; Joosten et al. 2005; Yamamoto et al. 2007)	PLVAP upregulation located around the area of ischemic damage (Shue et al. 2008)	Increased endothelial fenestration in NDP knockout mice Disrupted vascular integrity (Schafer et al. 2009)	Retinal neovascularization and hyperglycemia in Akimba (*Ins2*Akita*VEGF* ±) mouse model BRB loss, fluorescein leakage and focal angiogenesis in Ins2Akita (Akita) mice and VEGF photoreceptor-overexpressing trVEGF029 (Kimba) (Wisniewska-Kruk et al. 2014)
Characteristics	PLVAP colocalization with vascular endothelial markers: -cluster of differentiation 31 (CD31) -von Willebrand factor (Liu et al. 2010; Madden et al. 2004; Shue et al. 2008)	Nearly no zonula occludens-1 (ZO-1) and occludin expression in PLVAP-positive microvessels Spatial correlation of PLVAP with extravasated neutrophils in tissues (Mozer et al. 2010)	Chronic rejection in an allograft recipient due to double contouring of the glomerular capillaries formation of a basement membrane-like structure beneath the endothelium following endothelial injury (Ichimura et al. 2008; Joosten et al. 2005; Yamamoto et al. 2007)	48 h: ~ 17% of *CD31*-positive vessels expressed *PLVAP* 7 days: similar number of *PLVAP*-positive vessels and *CD31* expressing vessels (Shue et al. 2008)		
Specialties	Novel target for cancer therapy (Guo et al. 2016; Wang et al. 2014)			Novel target for cerebral edema therapy (Bosma et al. 2018)		Novel target for diabetic macular edema therapy (Bosma et al. 2018)

During inflammation, PLVAP was shown to be required for diapedesis of leukocytes into inflammation sites, to be important for the transcellular transmigration but not for adhesion and rolling of lymphoblasts, while having no effect on neutrophils transmigration (Elgueta et al. 2016; Keuschnigg et al. 2009). Furthermore, PLVAP was identified to be a receptor protein for the E-glycoprotein of Japanese Encephalitis Virus (JEV) in neurons. Accordingly, up- or downregulation of PLVAP led to an increase or a reduction of the viral load respectively (Mukherjee et al. 2018). Interestingly, in case of a SV40 infection, PLVAP is able to block low viral concentrations either by interfering at the level of the internalization pathway on the cell surface or afterwards (Tse et al. 2011). Thus, further investigations could reveal more possible interactions between PLVAP and virulence factors and deliver further insight in its role during inflammation and breakdown of the barrier endothelia.

Recently, a model was described, where the vascular barrier of the choroid plexus was closed by upregulation of Wnt/β-catenin signaling pathway in reaction to intestinal inflammation caused by bacteria-derived lipopolysaccharide, implicating involvement of the gut-brain vascular axis (Carloni et al. 2021). In this context, an increase in PLVAP levels was observed, which seemed to trigger the opening of the gut vascular barrier. Additionally, the authors observed a deficit in short-term memory and anxiety-like behavior, suggesting that PVB closure may correlate with mental deficits, mental symptoms like Inflammatory bowel disease, thus probably having its origin in a deregulated gut–brain vascular axis (Carloni et al. 2021). Besides this example, there might be other conditions, where there is a crosstalk between different vascular barriers that may also include a modulation of PLVAP expression. Moreover, other pathways than the Wnt/β-catenin signaling pathway could be involved in those progresses offering interesting new aspects for future research. To conclude, in both conditions, health and disease, PLVAP may maintain vascular integrity and homeostasis, a property that provides great potential for further research.

In the case of DME, osmotherapy, surgery, laser photocoagulation and steroids are common therapeutic approaches, while VEGF inhibitors have been established as innovative therapeutic tools, as they significantly improve the vision in DME patients (Rabinstein 2006, Wells et al. 2016). Furthermore, the utility of VEGF inhibitors is well documented in other ocular diseases as wet age-related macular degeneration, retinopathy of prematurity (ROP), retinal vein occlusion, and proliferative diabetic retinopathy (Braithwaite et al. 2014; Martinez-Zapata et al. 2014; Sankar et al. 2018; Solomon et al. 2019, Wells et al. 2016). But VEGF inhibitors carry some negative aspects, as VEGF regulates several cellular functions ensuring neuronal survival and function (Amato et al. 2016; Saint-Geniez et al. 2008; Sun et al. 2003). This appeared during systemic anti-VEGF therapies in cancer patients that displayed a higher risk of stroke and other arterial thromboembolic events (Scappaticci et al. 2007; Tolentino 2011). In this regard, increase of PLVAP mRNA levels in the retina is part of the pathological progression and neovascular leakage that could be improved by intravitreal injections of anti-PLVAP antibodies in cynomolgus monkeys (Nakagami et al. 2019). Therefore, PLVAP inhibition may be a promising new therapeutic approach to fight retinal diseases that deserves more attention in research.

## Further perspectives and concluding remarks

PLVAP is a central regulator of vascular permeability during embryogenesis, after birth and in mature endothelium. The diaphragms build by PLVAP provide a regulatory sieving function that needs to be further investigated to understand its role under pathological conditions, e.g. the molecular mechanisms that lead to barrier breakdown of the BBB and the BRB. In this context, upregulation of PLVAP was documented to be involved in cancer, traumatic spinal cord injury, acute ischemic brain disease, transplant glomerulopathy, Norrie disease and diabetic retinopathy. Additionally, an involvement of PLVAP during inflammatory challenge of the gut-brain vascular axis was suggested recently (Carloni et al. 2021). Furthermore, the potential of PLVAP as therapeutic target as a vehicle for targeted drug delivery needs to be further examined. The latter was observed in a study, where antibodies against PLVAP were conjugated to the therapeutic enzyme superoxide dismutase (SOD) to transport SOD to caveolae of endothelial cells of pulmonary vessels (Bosma et al. 2018; Shuvaev et al. 2018). This approach blocked lipopolysaccharide-induced pulmonary inflammation better than the conjugation of SOD to endothelial cells via CD31 and might thus offer interesting opportunities, e.g. the potential application at barrier endothelia as the BBB or BRB (Bosma et al. 2018; Shuvaev et al. 2018). Also, new techniques as single cell RNA sequencing might provide data with less contamination by other tissues and allow a deeper look into the biology of ECs with respect to their microenvironment and heterogeneity.

Finally, the role of different types of endothelia during organ function, and therefore also that of PLVAP, has to be considered and deserves more attention (Augustin and Koh 2017). In this regard, organ-specific endothelial cell lines are desirable, which maintain their typical expression levels of PLVAP and characteristic presentation of PLVAP-containing structures, as exemplified by the recently developed human choroid plexus endothelial cell line iHCPEnC (Muranyi et al. 2022). Assembly of those endothelial cell lines with other organ-specific cells, e.g. provided by organoids, would lead to the generation of in vitro models, which allow the targeted analysis of PLVAP function beyond its impact on endothelia only, in the context of organ functions (Zhang et al. 2021). Those models would provide an important step further for the development of strategies to target PLVAP function during treatment of disease.
